# Supplementary figures and images for: Parallel Evolution of Metazoan Mitochondrial Proteins
Source: Genome Biol Evol. 2017 Feb 14;9(5):1341–50. doi: 10.1093/gbe/evx025 (PMC5520408; doi:10.1093/gbe/evx025)

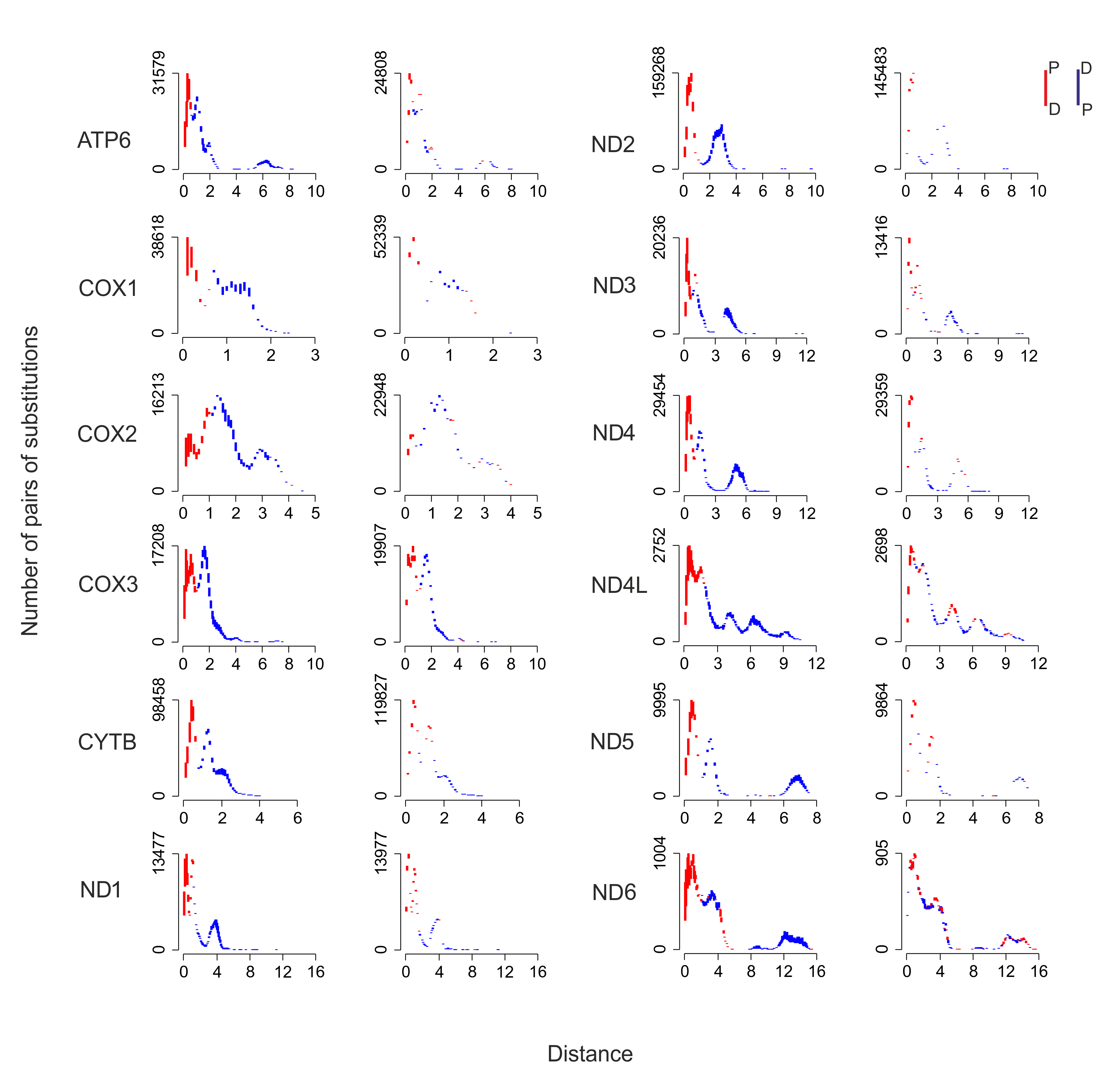

Supplement: Supplementary figure 1 [file suppl_figure_1.gif]

P/D ratio

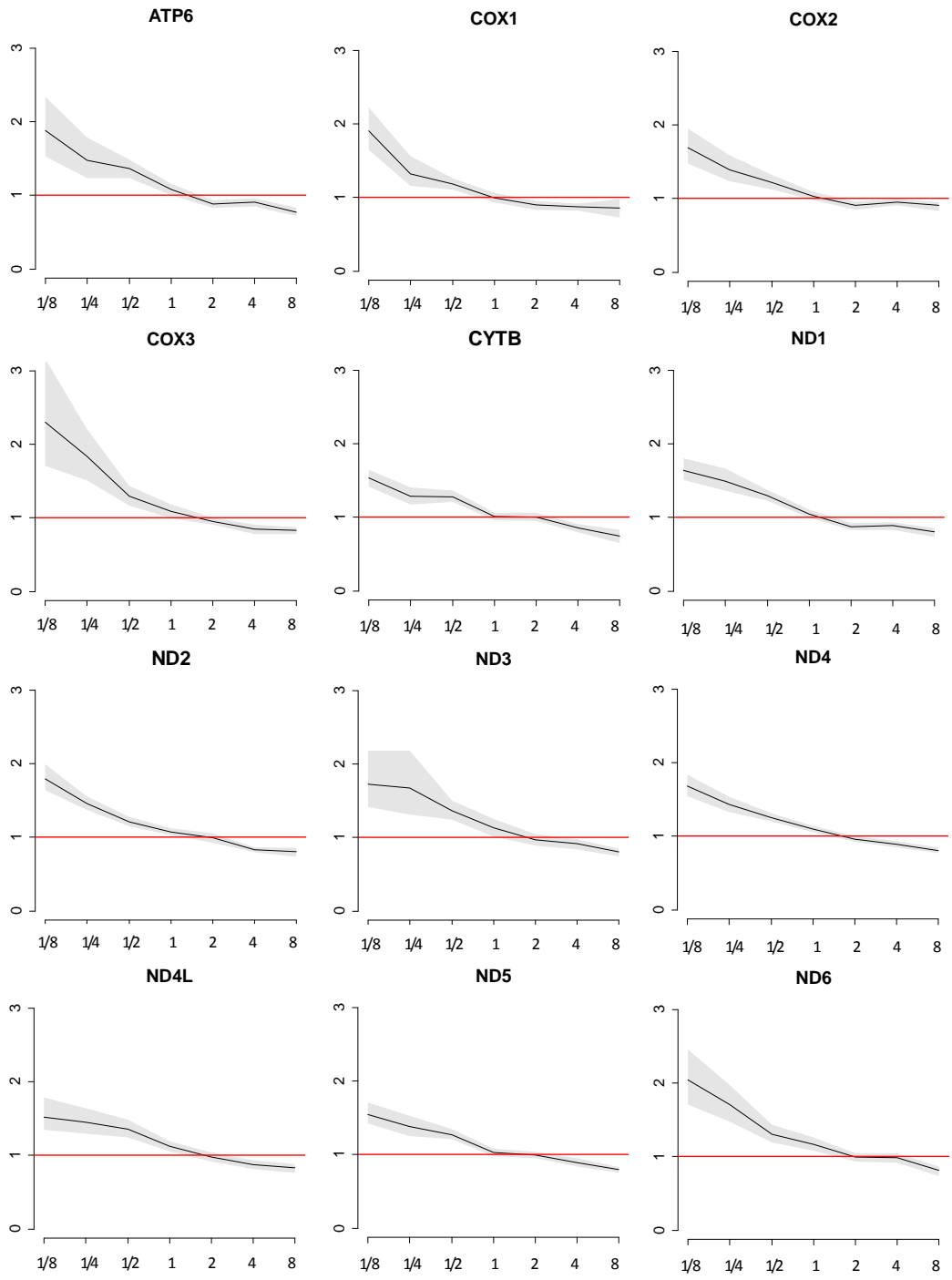

distance

Supplement: Supplementary figure 2 [file suppl_figure_2.pdf]

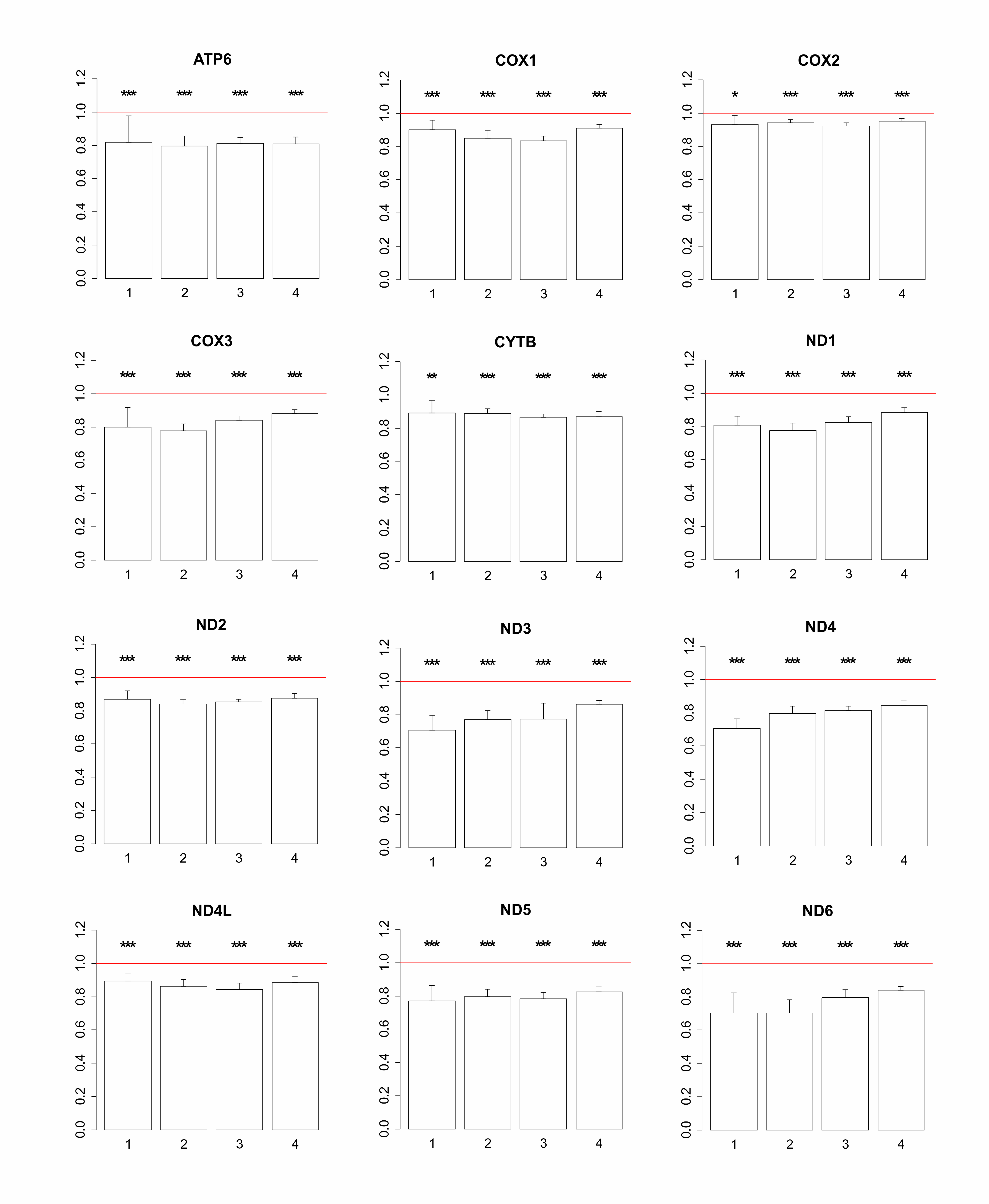

Supplement: Supplementary figure 3 [file suppl_figure_3.gif]

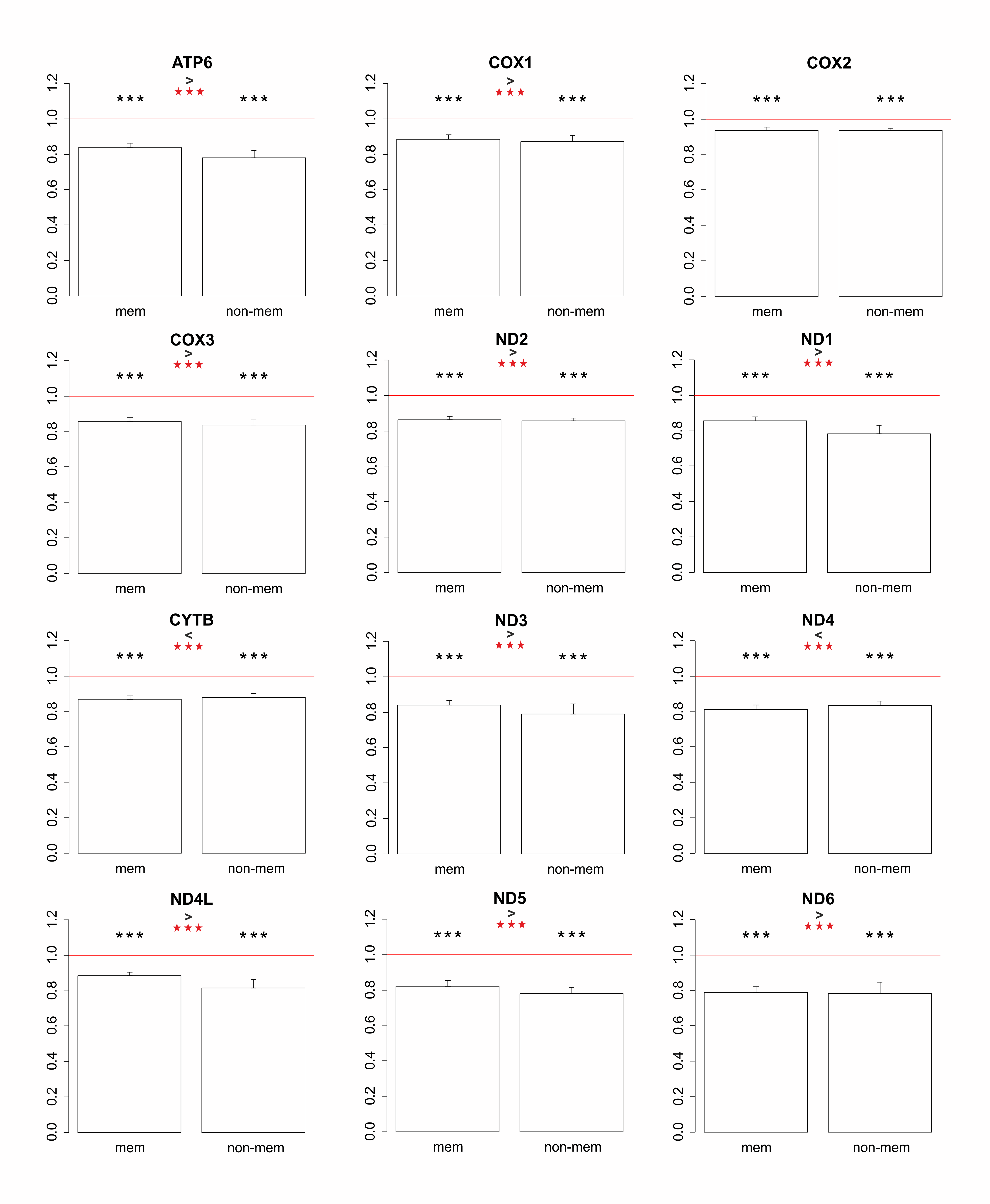

Supplement: Supplementary figure 4 [file suppl_figure_4.gif]

% of all unrelated  
branch pairs of the  
tree

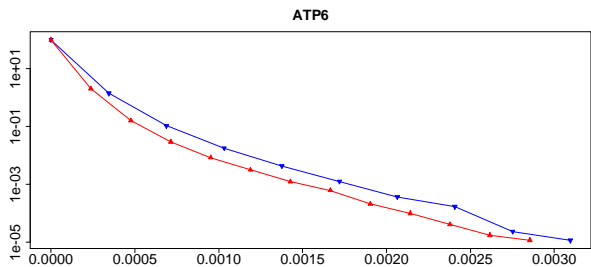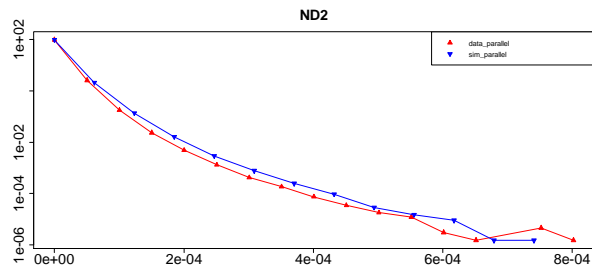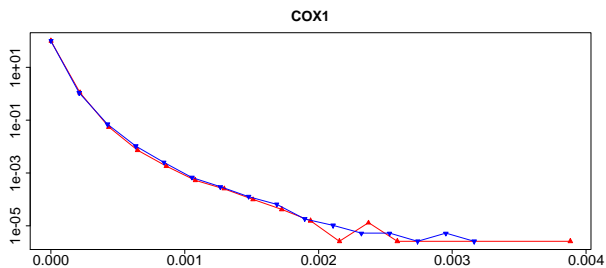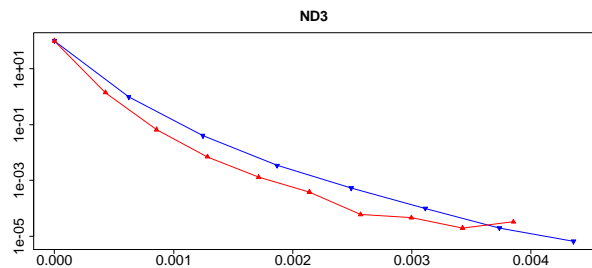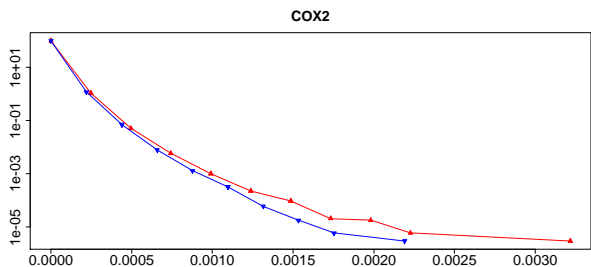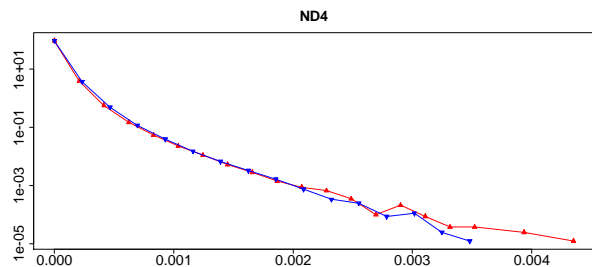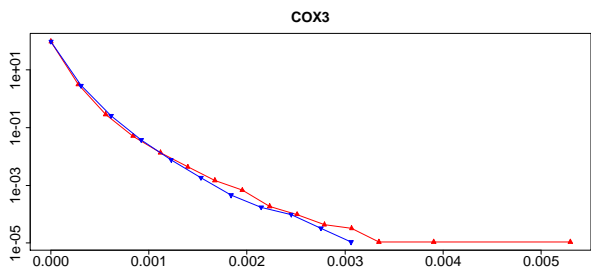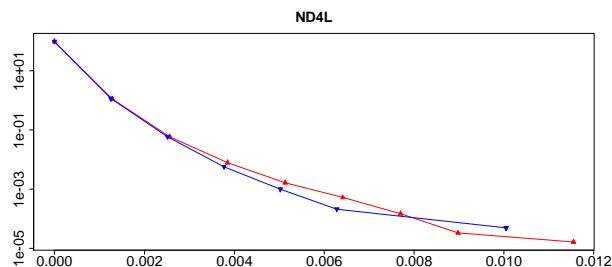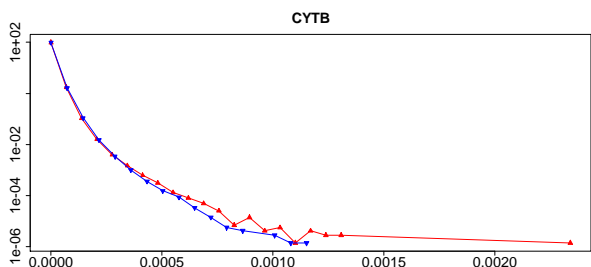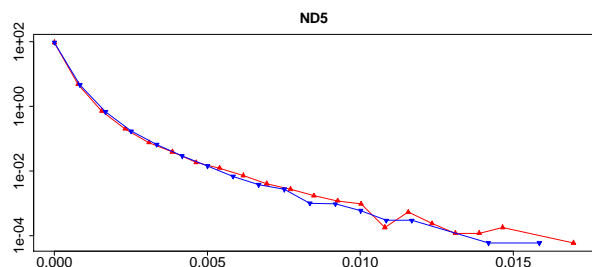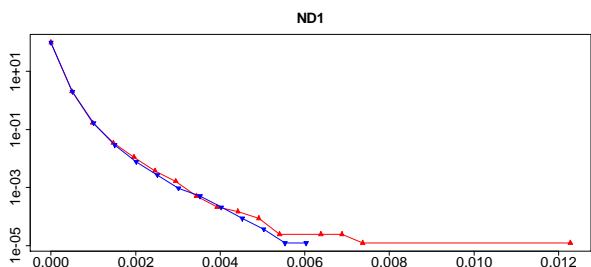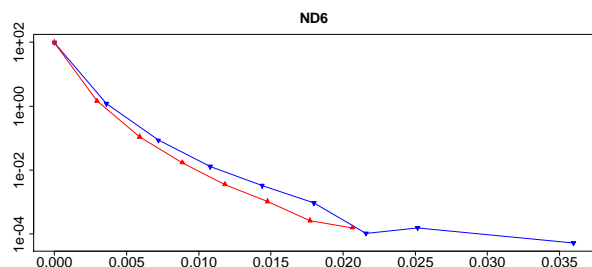

% of all substitution pairs  
from one branch pair

Supplement: Supplementary figure 5 [file suppl_figure_5.pdf]
