# Supplementary material for: Parallel Evolution of Metazoan Mitochondrial Proteins
Source: Genome Biol Evol. 2017 Feb 14;9(5):1341–50. doi: 10.1093/gbe/evx025 (PMC5520408; doi:10.1093/gbe/evx025)
Supplement: Supplementary table 1 [file suppl_table1.doc]

**Supplementary Table 1.**

Analysis of homoplasies in metazoan phylogenies.

| **Gene** | **Sites** | **Homoplasy informative sites*** |
| --- | --- | --- |
| ATP6 | 186 | 172 |
| COX1 | 404 | 317 |
| COX2 | 165 | 154 |
| COX3 | 198 | 173 |
| CYTB | 327 | 321 |
| ND1 | 253 | 227 |
| ND2 | 299 | 288 |
| ND3 | 94 | 87 |
| ND4 | 392 | 348 |
| ND4L | 82 | 79 |
| ND5 | 516 | 404 |
| ND6 | 119 | 111 |

* Homoplasy informative sites are those that carry at least one pair of parallel substitutions, and at least one pair of divergent substitutions, of the same amino acid.
